# Unraveling Th subsets: insights into their role in immune checkpoint inhibitor therapy

**DOI:** 10.1007/s13402-024-00992-0

**Published:** 2024-09-26

**Authors:** Monika Ryba-Stanisławowska

**Affiliations:** Department of Medical Immunology, Faculty of Medicine, Medical University of Gdańsk, Dębinki 1, Gdańsk, 80-211 Poland

**Keywords:** Th subsets, Anti-PD1, Anti-CTLA-4, ICI, Tumor immunity

## Abstract

T helper (Th) cell subsets play pivotal roles in regulating immune responses within the tumor microenvironment, influencing both tumor progression and anti-tumor immunity. Among these subsets, Th1 cells promote cytotoxic responses through the production of IFN-γ, while Th2 cells and regulatory T cells (Tregs) exert immunosuppressive effects that support tumor growth. Th9 and Th17 cells have context-dependent roles, contributing to both pro-inflammatory and regulatory processes in tumor immunity. Tumor antigen-specific T cells within the tumor microenvironment often exhibit a dysfunctional phenotype due to increased expression of inhibitory receptors such as CTLA-4 and PD-1, leading to reduced antitumor activity. Monoclonal antibodies that block these inhibitory signals—collectively known as immune checkpoint inhibitors (ICIs)—can reactivate these T cells, enhancing their ability to target and destroy cancer cells. Recent advancements have highlighted the critical role of T helper subsets in modulating responses to ICIs, with their interactions remaining a focus of ongoing research. Both positive and negative effects of ICIs have been reported in relation to Th cell subsets, with some effects depending on the type of tumor microenvironment. This review summarizes the crucial roles of different T helper cell subsets in tumor immunity and their complex relationship with immune checkpoint inhibitor therapy.

## Immune checkpoint receptors as targets for cancer immunotherapy

Cancer immunotherapy aims to adjust the patient’s immune system to trigger an anti-tumor response. Tumors create an immunosuppressive microenvironment that inhibits the immune system’s natural ability to recognize and destroy transformed cells. This is accomplished via several mechanisms, including the recruitment of immunosuppressive cells, exclusion of T cells, and activation of immune checkpoint pathways [1]. These pathways involve various inhibitory receptors, the most well-known and studied of which are cytotoxic T lymphocyte-associated antigen 4 (CTLA-4), which binds to B7-1 (CD80) and B7-2 (CD86) molecules, and programmed death 1 (PD-1), which engages with the PD-L1 (B7-H1) and PD-L2 ligands [1, 2].

CTLA-4, the primary immune checkpoint receptor to be therapeutically targeted, is present exclusively on T cells, where its main function is to control the intensity of T cell activation in the initial phases [2]. Essentially, CTLA-4 counteracts the function of CD28, a co-stimulatory receptor on T cells [3]. It functions as a signal “silencer” to maintain balance and prevent excessive activation of T cells [2]. The second crucial immune checkpoint receptor, PD-1, restricts the function of T cells within peripheral tissues during an inflammatory response to infection [2, 4]. Its role also extends to limiting autoimmunity [2, 4]. Activation of PD-1 occurs when it binds to either of its two known ligands, PD-L1 or PD-L2. Both ligands are found on the surface of antigen-presenting cells (APCs), such as dendritic cells, and PD-L1 is also expressed on various other cell types, including tumor cells. PD-L1 specifically contributes to tumor immune modulation and has a suppressive effect [5–7].

T cells in tumor microenvironment frequently exhibit a dysfunctional (exhausted) phenotype, marked by diminished effector function and elevated levels of CTLA-4 and PD-1 expression [1–4]. The increased expression of CTLA-4 and PD-1 is likely due to prolonged antigen stimulation, oncogenic signaling, and inflammation within the tumor microenvironment [1, 2, 8–5, 10]. Some tumors, such as melanomas, have been categorized into two groups based on the presence or absence of inflammation, as determined by the expression levels of various inflammatory genes, including those related to interferon pathways [10]. In a study of melanoma, Taube et al. observed a significant association between cell surface PD-L1 expression on tumor cells and immune activity, specifically lymphocytic infiltration and intratumoral interferon γ (IFN-γ) expression. This suggests that PD-L1-positive tumors may exhibit a more active immune response characterized by IFN-γ production. This correlation was consistently observed across different melanoma tumors studied. Within individual tumors that expressed PD-L1, these associations were also consistently observed across different regions of the tumor [5]. These findings imply the existence of a negative feedback mechanism wherein IFN-γ triggers the expression of PD-L1, which subsequently inhibits the function of PD-1+ T cells [2, 5].

**Fig. 1:**
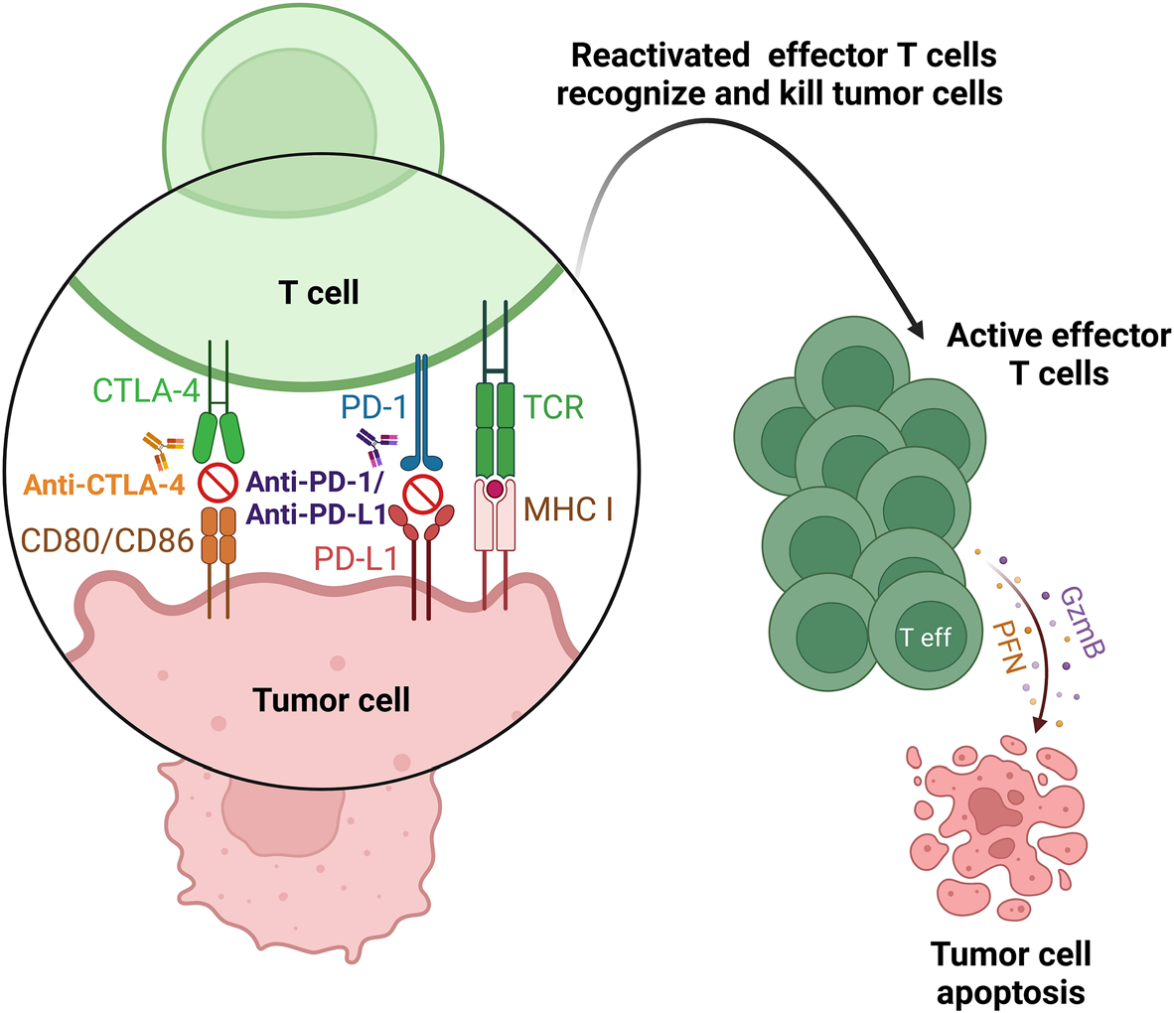
Mechanism of action of immune checkpoint inhibitors. Tumor cells use CD80/CD86 to bind CTLA-4 and PD-L1 to bind PD-1 on T cells, inhibiting antitumor T cell activity. Blocking CD80/CD86 from binding to CTLA-4 with anti-CTLA-4 antibodies and PD-L1 from binding to PD-1 with anti-PD-1 antibodies or directly blocking PD-L1 with anti-PD-L1 antibodies reactivates T cells, enabling them to kill tumor cells. *Abbreviations TCR* t cell receptor, *MHC I* major histocompatibility complex I, *CTLA-4* cytotoxic T-lymphocyte-associated antigen 4, *PD-1* programmed death 1, *PD-L1* programmed death ligand 1, *Teff* effector T cell, *GZMB* granzyme B, *PFN* perforin. The figure was prepared using Biorender

Inhibitory signals on T cells can be blocked by checkpoint inhibitors (ICIs), thereby reactivating them to attack cancer cells more effectively (Fig. 1). The approved immunotherapeutic strategy involves the use of monoclonal antibodies aimed at these immune checkpoints [2, 6, 7, 11]. Checkpoint inhibitors used in cancer immunotherapy to block immune checkpoints and reactivate T cells, allowing them to participate in the anti-tumor immune response, include antibodies against:

- CTLA-4, such as Ipilimumab (Yervoy) and Tremelimumab (Imjuno)
- PD-1, such as Pembrolizumab (Keytruda), Nivolumab (Opdivo) and Cemiplimab (Libtayo)
- PD-L1, such as Atezolizumab (Tecentriq) and Durvalumab (Imfinzi)

The treatment of cancer patients with immune checkpoint inhibitors (such as anti-CTLA-4, anti-PD-1, anti-PD-L1, or combinations of anti-PD-1/PD-L1 with anti-CTLA-4) has marked a major breakthrough in oncology in recent years. This form of immunotherapy represents a significant advancement in cancer treatment. ICIs have greatly improved treatment outcomes and prognoses for patients with various types of malignancies, including melanoma, lung cancer, urothelial carcinoma, renal cell carcinoma, hepatocellular carcinoma, cutaneous squamous cell carcinoma, colorectal cancer, head and neck cancers, and neoplasms of the lymphatic system such as Hodgkin’s lymphoma [7, 11–13].

## T helper lymphocyte subsets in tumor immunity and their association with immune checkpoint inhibitor therapy

**Fig. 2:**
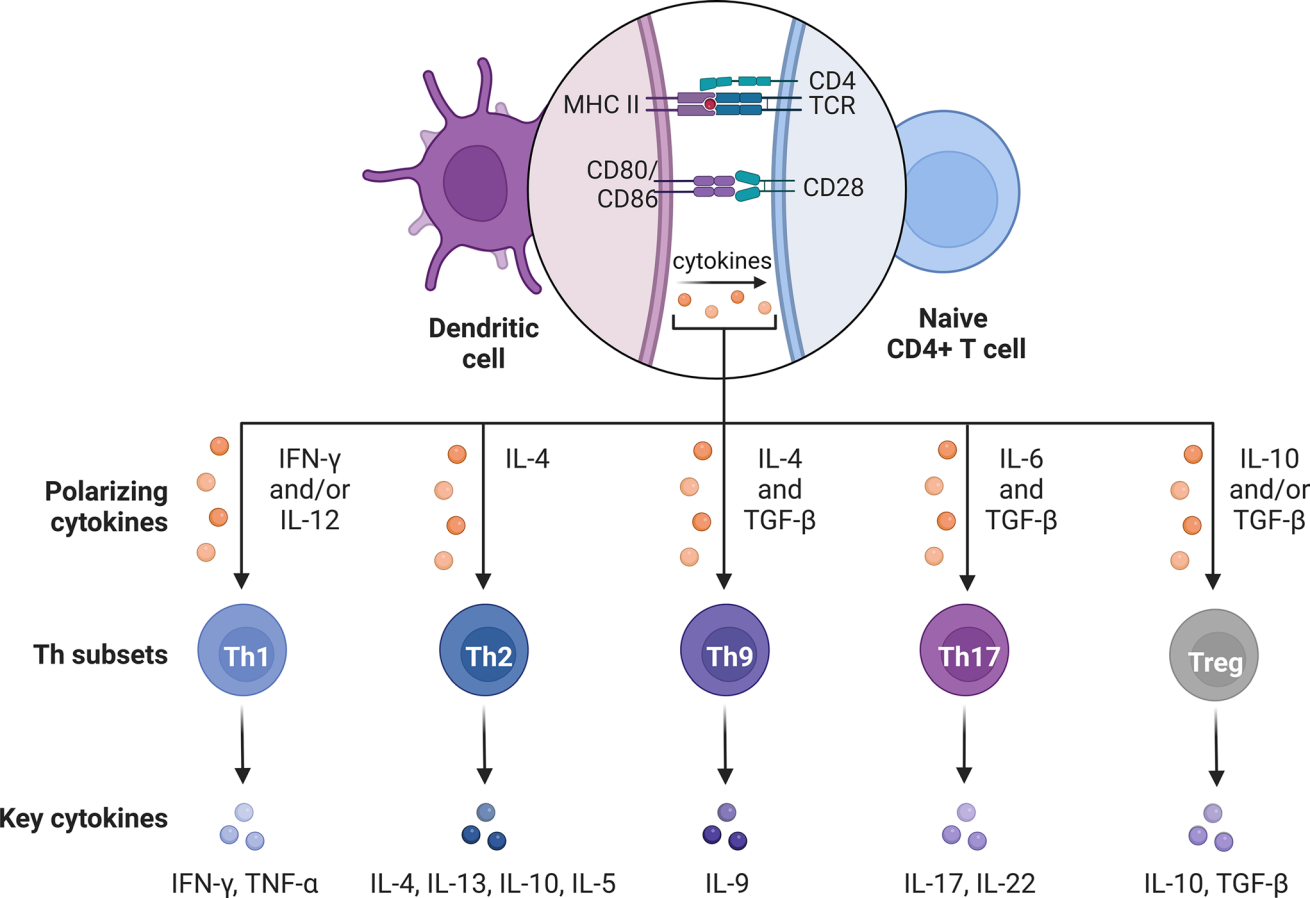
Polarization of naive CD4+ T cell into different effector cell types. During TCR activation under particular polarizing conditions, naive CD4+ T cells differentiate into one of several lineages of helper T cells. Based on the profile of cytokines they produce, these cells constitute functionally distinct subsets with different properties. *Abbreviations TCR* t cell receptor, *MHC II* major histocompatibility complex II, *Th* t helper, *IL-* interleukin-, *IFN-γ* interferon γ, *TNF-α* tumor necrosis factor α, *TGF-β* transforming growth factor β. The figure was prepared using Biorender

In the context of tumor immunity, various cell populations, including T helper lymphocytes (Th), can influence the anti-tumor immune response and impact cancer patient outcomes. Naive CD4+ T cells polarize into distinct effector subsets when exposed to specific cytokines, often referred to as polarizing cytokines, within their environment. Upon activation, these cells mature into effector T helper cells with diverse cytokine secretion patterns and functions. These subsets of effector Th cells exhibit distinct cytokine profiles (Fig. 2) and play specific roles in coordinating and regulating the immune response. Their unique characteristics are tailored to effectively address various immune challenges.

Subpopulations of T helper lymphocytes are being studied for their roles and effects on immune responses associated with tumors and their treatment with immune checkpoint inhibitors.

### T helper type 1 cells

T helper type 1 cells (Th1) are a subset of CD4+ effector T cells that differentiate under the influence of interleukin-12 (IL-12) and/or interferon γ (IFN-γ). They play a crucial role in initiating cellular immune responses and defence against intracellular pathogens [14, 15]. Th1 cells primarily secrete type 1 cytokines, with key examples including IFN-γ and tumor necrosis factor α (TNF-α). These cytokines stimulate macrophages through the classical activation pathway, leading to the production of nitric oxide (NO), reactive oxygen species (ROS), and other proinflammatory factors [14]. Elevated Th1 responses have been linked to autoimmune diseases such as rheumatoid arthritis (RA), multiple sclerosis (MS), inflammatory bowel disease (IBD), and type 1 diabetes (T1D) [14].

Within the framework of antitumour immune responses, Th1 cells and IFN-γ are crucial effectors [16–18]. IFN-γ exhibits proapoptotic, tumouricidal, and antiangiogenic effects. It activates natural killer (NK) cells and cytotoxic T lymphocytes (CTLs) in the antitumour immune response. Th1 cells enhance CTL effectiveness by modifying their gene expression profile, reducing inhibitory signals, and providing additional activation signals through interactions with dendritic cells (DCs) and CD27, a key molecule on CTLs that supports their activation, survival, differentiation, and effector functions [19]. This collaboration optimises the immune response against cancer cells. Furthermore, IFN-γ enhances the expression of major histocompatibility complex (MHC) molecules, facilitating the presentation of tumour antigens, and it stimulates macrophages towards a proinflammatory, tumouricidal M1 phenotype [20]. IFN-γ also acts as a cytotoxic cytokine, working together with other cytotoxic factors like granzyme B and perforin to initiate apoptosis in tumour cells [20–22]. Paradoxically, however, IFN-γ may promote the growth and progression of certain cancers [17, 22, 23]. For instance, IFN-γ facilitates the synthesis of indoleamine-2,3-dioxygenase (IDO), an enzyme involved in immune regulation, thereby stimulating other immune-suppressive mechanisms [23–26]. Studies have also shown that IFN-γ recruits myeloid-derived suppressor cells (MDSCs) to the tumour microenvironment, which subsequently inhibit T cell responses [27, 28].The activity of IFN-γ within the tumor microenvironment might negatively impact the immune system’s ability to fight the tumor. Specifically, IFN-γ may alter the balance or function of T cell populations in a way that weakens the overall anti-tumour immune response.

In addition, IFN-γ may affect the effectiveness of anti-PD-1 antibody therapy, particularly as it can induce the expression of PD-1 ligands on the surface of some tumour cells, including those in ovarian cancer, potentially promoting tumour progression [29]. Studies have demonstrated that prolonged IFN-γ signalling may promote resistance to immune checkpoint inhibitor (ICI) therapy, as well as to the combination of ICI with radiation [30–32]. Conversely, research by Ayers et al. found that IFN-γ signalling within the tumour microenvironment is a common feature of tumors that show positive responses to PD-1 blockade therapy with Pembrolizumab. The study demonstrated that activation of IFN-γ-responsive genes, which are involved in processes such as antigen presentation, cytotoxic T cell responses, and chemokine expression, was critical for achieving clinical effectiveness in patients across multiple cancer types, including melanoma, bladder, gastric, ovarian, and colorectal cancer [33]. This suggests that IFN-γ-related pathways play an important role in the success of PD-1 blockade immunotherapy in various cancers.

Besides IFN-γ, the Th1-related cytokine IL-12 plays a crucial role in the immune response overall and is particularly significant for ICI therapy. Garris et al. demonstrated that successful anti-PD-1 therapy in mice depends on the production of IL-12 by intratumoral dendritic cells [34]. These dendritic cells did not bind anti-PD-1; instead, they produced IL-12 in response to IFN-γ secreted by nearby anti-PD-1-activated CD8+ T cells. The IL-12 produced by the dendritic cells then enhanced the antitumor activity of T cells [34]. The authors also found that IL-12 was able to induce cytolytic activity of tumor infiltrating T lymphocytes in patients with melanoma [34]. Additional experiments have shown that intratumoral administration of IL-12 upregulates the expression of key genes implicated in cytolysis within tumors [33, 35]. Tumors expressing these genes, including CD2, CD3E, CD247, GZMA, GZMH, GZMK, NKG7, and PRF1, are associated with antitumour immune responses and are more likely to respond to anti-PD-1 immunotherapy [35, 36]. This connection underscores the importance of these genes in predicting and influencing the efficacy of PD-1 blockade in cancer treatment.

A significant marker of the Th1-associated antitumour immune response is the inducible co-stimulator (ICOS) [37, 38]. This was first identified in a clinical trial evaluating the use of Ipilimumab (an anti-CTLA-4 antibody) in bladder cancer patients [39]. The authors observed that CD4+ T cells from both blood and tumour tissue of all treated patients expressed increased levels of ICOS and produced IFN-γ. These CD4+ ICOShigh IFN-γ-producing T cells demonstrated the ability to recognise tumour antigens, and their proliferation increased the ratio of effector T cells (Teffs) to regulatory T cells (Tregs) in both peripheral blood and tumour tissue [39]. Similar observations were made by Chen et al. in the case of prostate cancer [40]. The authors demonstrated that CD4+ ICOS+ T cells constitute a subset of effector Th1 cells specific to tumour antigens. Moreover, the absence of ICOS led to a significant reduction in anti-tumour T-cell responses triggered by the anti-CTLA-4 antibody, thereby hindering tumour rejection [41]. Additionally, increased ICOS levels were shown to correlate with elevated expression of the Th1 transcription factor T-bet after treatment with the anti-CTLA-4 antibody [42]. These data confirm the essential role of the ICOS/ICOSL pathway in maximising the therapeutic impact of anti-CTLA-4 antibody therapy on Th1 lymphocytes, suggesting that this pathway could be a promising target for future combined approaches aimed at enhancing the effectiveness of anti-CTLA-4 treatment.

### T helper type 2 cells

T helper type 2 (Th2) cells are a distinct lineage of CD4+ effector T cells that differentiate in response to IL-4 and secrete type 2 cytokines, such as IL-4, IL-5, and IL-13 [14, 43]. These cells are crucial for humoral immunity and play a key role in coordinating the immune response against large extracellular pathogens. Excessive Th2 immune responses have been implicated in the development of chronic allergic inflammation and asthma [43].

In the context of tumour immunity, Th2 cells are thought to promote tumour growth through their angiogenic activity and inhibitory effects on cell-mediated immunity, mediated by Th2 cytokines [44, 45]. However, some studies demonstrate the anti-tumour activity of Th2 lymphocytes, mainly through their effects on tumour-infiltrating granulocytes, such as eosinophils, which can directly kill tumour cells [46]. Additionally, tumour-specific antibodies may induce antibody-dependent cytotoxicity (ADCC) and tumour clearance [47]. Unlike type-1-mediated anti-tumour immunity, which triggers tumour cell apoptosis, type-2-mediated immunity appears to lead to tumour necrosis [46]. For instance, IL-4 has been reported to have anti-angiogenic properties, potentially inducing necrosis in solid tumours once they reach a certain size [48]. Similarly, IL-4 expression in glioma cells has been associated with reduced vascularisation and tumour growth [49], possibly influenced by tumour-associated fibroblasts (TAFs), which inhibit angiogenesis when stimulated by IL-4 [50]. On the other hand, IL-4 has also been reported to inhibit cell apoptosis, potentially promoting tumour growth [51]. While IL-4 is recognised as the most crucial Th2 cytokine, IL-13 is another cytokine upregulated during Th2-mediated immune responses [43]. IL-4’s anti-tumour effects are primarily mediated through eosinophils and macrophages, whereas IL-13’s anti-tumour activity is reported to be mediated by neutrophils and macrophages [52]. IL-13 has also been reported to suppress anti-tumour immunity by inhibiting IFN-γ secretion and the activity of cytotoxic T lymphocytes [53]. The next cytokine produced by Th2 lymphocytes, IL-10, is primarily known for its ability to downregulate anti-tumour immunity [54]. However, increasing evidence suggests that IL-10 is not consistently immunosuppressive and does not always promote tumour immune evasion [55]. For example, IL-10 has been reported to have anti-angiogenic properties [56, 57], which may help inhibit tumour growth. Therefore, IL-10 exhibits complex pleiotropic effects on anti-tumour immunity, with the potential to either enhance or reduce tumour immunogenicity.

Data on the impact of immune checkpoint inhibitors on Th2 cells is limited; however, some studies suggest a potential link between ICI treatment and Th2 immunity. Blomberg et al. analysed blood and tumors from metastatic breast cancer patients undergoing treatment with checkpoint inhibitors, complemented by studies in mouse models of breast cancer [58]. They observed an increase in both systemic and intratumoral eosinophils in patients and mice that responded to ICI [58]. ICI treatment promoted the production of IL-5 by Th2 lymphocytes, which subsequently stimulated increased eosinophil production in the bone marrow, leading to a systemic expansion of these cells [58]. In responders, ICI treatment resulted in increased expression of eosinophil-related genes within tumours, a change not observed in non-responders. Depleting eosinophils inhibited both CD8+ T cell activation and the response to ICI. IL-5-producing Th2 cells have been shown to drive eosinophil production and their systemic expansion following ICI treatment [58]. It has been proposed that eosinophils induced by ICI treatment may directly target tumours, exerting tumouricidal effects, or they may enhance anti-tumour immunity by altering tumour vasculature or modifying the immune environment [58]. In the same study, Blomberg et al. demonstrated that eosinophils activated CD8+ cytotoxic T lymphocytes in tumours responding to therapy with checkpoint inhibitors [58]. Additionally, the authors observed higher levels of genes typically associated with eosinophils, as well as genes associated with CD8+ T cells and IFN-γ. This correlation was specifically noted in metastatic breast cancer lesions of patients who responded to ICI [59]. These data suggest that eosinophils, as part of Th2 immunity, are influenced by ICI treatment and have the potential to significantly impact local immunity and tissue remodelling in certain cancers that respond to checkpoint inhibitor therapy. In other studies exploring the impact of ICIs on Th2 cells circulating in peripheral blood, Saito et al. investigated changes in PBMC profiles during treatment with Nivolumab (anti-PD-1 antibody). Initially, their analysis indicated a dominance of Th2 immune responses. However, in a positive case where the patient responded well to the anti-PD-1 antibody, there was a noticeable shift towards a Th1/T-cytotoxic immune profile following drug administration. This shift suggests a favourable change towards a more cytotoxic immune response, which is often associated with better outcomes in immunotherapy [60]. In another study, investigators explored variations in PD-1/PD-L1 expression among different lymphocyte subsets in peripheral blood using a publicly available dataset [61]. They observed notably elevated PD-1 expression in CD8+ and CD4+ T cells, with B cells also showing high PD-1 expression compared to NK cells. This led them to speculate that melanoma might induce an immunosuppressive effect on immune cells throughout the body, particularly those in peripheral blood. To test this hypothesis, they compared the immune profiles of healthy individuals with those of melanoma patients. However, they found no significant difference in the levels of Th2 cytokines in the peripheral blood between the two groups [62]. Furthermore, analysis of peripheral blood T cell status in patients treated with Nivolumab showed no differences in Th2 cells and their cytokines compared to levels before treatment [61]. These findings suggest that certain types of tumours, such as melanoma, may not induce systemic immunosuppression solely mediated by the tumour itself [61].

### T helper 9 cells

A subset closely related to Th2 lymphocytes, with notable plasticity between the two cell types, is T helper 9 cells (Th9) [62]. Naive Th cells differentiate into Th9 cells under the influence of IL-4 and transforming growth factor β (TGF-β) [62–65]. Th9 cells are crucial for defending the host against parasitic infections. However, they can also have detrimental effects, such as inducing chronic allergic inflammation, airway remodelling, and the development of autoimmune diseases [65].

Th9 cells and their primary cytokine, IL-9, can exhibit either pro-tumor or anti-tumor activity depending on the type of tumour and the surrounding microenvironment [44, 66–69]. The antitumor activity of Th9 cells appears to be more pronounced in solid tumours, as their tumouricidal effects have been demonstrated in melanoma, lung adenocarcinoma, colon cancer, and breast cancer [68–71]. Th9 cells were found to have cytotoxic activity comparable to Th1 cells and were also less exhausted [72]. Conversely, IL-9 has been associated with the progression of certain cancers, particularly haematologic malignancies [73–76]. High IL-9 gene expression is notably observed in patients with haematologic malignancies such as adult T-cell leukaemia (ATCL), anaplastic large-cell lymphoma (ALCL), Hodgkin lymphoma (HD), and NKT-cell lymphoma [44, 65]. Th9 cells have been suggested to potentially facilitate tumour growth in hepatocellular carcinoma, as they are more abundant in peritumour and tumour tissues compared to unaffected tissues. Additionally, patients with higher levels of Th9 cell infiltration have shown shorter disease-free survival periods [44]. Studies suggest that IL-9 promotes tumour growth by both inhibiting apoptosis and enhancing the proliferation of malignant cells [76, 77]. The reasons for the varying effects of Th9 cells on different cancers are not fully understood, but researchers suggest this may be related to the differential expression of the IL-9 receptor (IL-9R) on various cancer cell types [65].

The interplay between Th9 cells and immune checkpoint inhibitor (ICI) therapy remains an active area of investigation. Researchers are exploring how Th9 cells influence the efficacy of ICI treatment, with the goal of uncovering their specific roles in modulating the anti-tumour immune response. Studies by Wang et al. have shown that Th9 cells can be regulated through the PD-1/PD-L1 pathway and can promote the expansion of CD8+ T cells [78]. These findings suggest that Th9 cells may enhance the anti-tumour activity of ICI therapy by contributing to the activation of cytotoxic CD8+ T lymphocytes in human colorectal cancer [78]. Moreover, research has demonstrated that the infiltration of Th9 cells into tumours correlates with an increased frequency of cytotoxic T lymphocytes (CTLs) [78]. Th9 lymphocytes within malignant lesions were found to express high levels of the PD-1 receptor [78]. Inhibition of PD-L1 led to decreased IL-9 production, a suppression that was reversed by anti-PD-1 blockade. This phenomenon was observed not only in human colorectal cancer but also in mouse hepatocellular carcinoma [78]. In another study using a mouse model of lung cancer, Feng et al. demonstrated that IL-9 inhibited tumour growth by enhancing the expression of major MHC class I molecules on tumour cells, thereby promoting the immune response of cytotoxic T cells [79]. The authors suggested IL-9 as a potential therapeutic agent to be combined with PD-1 blockade for treating lung cancer immunotherapeutically [79].

The Th9 subset shows particularly strong anti-tumour activity in melanoma [66]. Nonomura et al. found that in melanoma patients who responded to treatment with Nivolumab (anti-PD-1 antibody), Th9 cells in peripheral blood were significantly elevated [80]. Additionally, responders had notably higher serum concentrations of TGF-β, which supports Th9 development, compared to non-responders. In vitro experiments demonstrated that the anti-PD-1 antibody enhanced Th9 differentiation. Analysis of human melanoma lesions revealed that Th9 cells were located in close proximity to CD8+ T cells [80]. Further studies using a melanoma mouse model showed that an antibody against IL-9 reduced the production of granzyme B and perforin by CD8+ T cells [80]. Moreover, in vitro studies indicated that IL-9 increased the cytotoxicity of tumour-specific CD8+ T cells in mice [80]. The authors proposed that Th9 cells could play a crucial role in the anti-tumour immune response in melanoma, and that treatment with the anti-PD-1 antibody might enhance this response by stimulating Th9 differentiation in melanoma patients who respond to Nivolumab [80]. Nonomura et al. suggested that the Th9 cell subset could serve as a biomarker for predicting a positive response to Nivolumab in melanoma patients [80]. In turn, in muscle-invasive bladder cancer (MIBC), the presence of IL-9-producing Th cells in the tumor microenvironment was correlated with an exhausted phenotype of CD8+ T cells [81]. It was demonstrated that Th9 cell infiltration within tumor tissue promoted an immunoevasive environment, characterized by reduced cytotoxic activity of CD8+ T cells, which expressed high levels of inhibitory receptors, including PD-1, in the presence of IL-9-producing Th cells [81]. Due to the increased PD-1 expression on CD8+ T cells alongside elevated infiltration of IL-9+ Th cells, the authors investigated whether IL-9-producing Th cells could predict the efficacy of anti-PD-1 antibody immunotherapy. Remarkably, they observed that, following administration of Nivolumab, the production of granzymes and perforin by CD8+ T cells, as well as their proliferative ability, only increased in the group with high levels of IL-9+ T cells. Furthermore, the proliferation of tumor cells was suppressed, and a greater number of apoptotic tumor cells were detected after Nivolumab treatment exclusively in the group of patients with high levels of IL-9+ T cells. These findings led the authors to suggest that patients with MIBC who have higher intratumoral infiltration of IL-9-producing Th cells might experience a more favorable response to anti-PD-1 antibody immunotherapy [81].

### T helper type 17 cells

T helper type 17 (Th17) cells were identified in 2005 as cells that secrete high levels of IL-17 and other inflammatory cytokines [82, 83]. They differentiate in response to TGF-β and IL-6 and play a role in the immune response against extracellular bacteria and fungi. The cytokines produced by Th17 cells induce resident cells to secrete chemokines, which attract neutrophils and macrophages to inflammatory sites. These recruited cells, in turn, release additional cytokines and proteases, amplifying the inflammatory immune response [84]. Although Th17 cytokines are essential for clearing harmful microbes, their sustained release can lead to chronic inflammation and contribute to various inflammatory and autoimmune diseases such as RA, MS, and IBD [84].

Th17 cells and IL-17 have been detected in serum, peripheral blood mononuclear cells, tumor tissues, and tumor-associated fluids across various types of cancer [85]. Studies have found evidence supporting both cancer-promoting and cancer-inhibiting roles for Th17 cells, with these roles varying depending on the type of cancer and the context of the tumor microenvironment [44, 85, 86]. The pro-tumorigenic function of Th17 cells in carcinogenesis is frequently associated with chronic inflammation. Excessive inflammation caused by Th17 cells is known to contribute significantly to the pathogenesis of several inflammation-associated cancers, including colon, lung, and liver cancers [87]. Chronic inflammation plays a dual role: it not only promotes cancer development but also supports the differentiation and activity of Th17 cells. In certain types of tumors, Th17 cell frequencies in both peripheral blood and malignant tissue have been observed to be higher compared to those found in healthy individuals [44, 88–92]. Peng et al. discovered that the frequency of circulating Th17 cells in renal cell carcinoma (RCC) patients was elevated and correlated with both the tumor’s stage and grade [89]. Furthermore, in solid tumors such as cutaneous squamous cell carcinoma and head and neck squamous cell carcinoma, elevated levels of intratumoral or serum IL-17 have been associated with poor prognosis [85, 92–94]. Similarly, in lung cancers, especially those resulting from cigarette smoking, whose onset is associated with inflammation, Th17 cells play a significant role [95]. They exacerbate chronic tissue inflammation and accelerate disease onset by attracting myeloid cells [96]. Most studies on non-small cell lung cancers have found that higher levels of IL-17 are associated with poorer outcomes [85]. However, some research has shown that Th17 cells can also exhibit anti-tumor effects. These effects include the direct secretion of IFN-γ [44] and the indirect recruitment of dendritic cells, NK cells, and CD8+ cytotoxic T lymphocytes to tumor sites. This recruitment subsequently enhances specific immune responses against tumor cells [97]. The Th17 subset expressing the CD26 molecule has been found to inhibit tumor growth [98, 99]. In addition to producing IL-17, these cells exhibit potent cytotoxic activity, including the expression of Granzyme B and CD107a, a marker of cytotoxic cell activation and degranulation. Furthermore, these cells demonstrate resistance to apoptosis [99].

With respect to ICI therapy, the role of Th17 cells remains not fully uncovered, with both positive and negative effects reported. While ICI therapy generally expands the Th1 population across different tumor types, tissue-specific conditions or variations in the tumor microenvironment may influence the differentiation of CD4+ T cells towards other Th subsets. For instance, a study by Jiao et al. demonstrated that in patients with metastatic prostate cancer, Ipilimumab enhanced the Th1 subset in soft tissue metastases but led to an expansion of Th17 cells in bone metastases [100]. The cytokine environment in bone metastases is characterized by elevated levels of TGF-β due to metastasis, coupled with naturally high levels of IL-6 from the bone marrow. These elevated levels of TGF-β and IL-6 drive the differentiation of CD4+ T cells towards the Th17 lineage instead of the Th1 lineage [100]. This example demonstrates how the specific cytokine environment within the tumor can influence the effectiveness of immune checkpoint therapy by directing CD4+ T cells away from a Th1 phenotype towards Th17 cells, which may compromise anti-cancer immunity. Krieg et al. studied immune cell subsets in the peripheral blood of stage IV melanoma patients treated with PD-1 inhibitors [101]. They found that responders had increased frequencies of IL-17 A among T cells after therapy, along with elevated levels of other T cell activation markers such as IFN-γ, PD-1, and granzyme B [101]. Additionally, recent studies by Váraljai et al. on BRAFV600-mutated melanomas and their response to immune checkpoint inhibition, particularly dual therapy targeting both PD-1 and CTLA-4, have demonstrated that IL-17 supports the anti-tumor effects of combined ICI therapy [102]. The authors demonstrated that BRAFV600-mutated tumors exhibited elevated presence or activity of genes typically associated with Th17 cells, correlating with clinical responses to combined immune checkpoint inhibition therapy involving PD-1 and CTLA-4 inhibitors [102]. High levels of Th17 gene expression were linked to increased infiltration of immune cells, including T cells and neutrophils, into tumors, thereby enhancing anti-tumor immune responses. Consequently, Th17 gene expression patterns are proposed as biomarkers to predict the effectiveness of immunotherapies, particularly those combining PD-1 and CTLA-4 inhibitors [102]. Th17 cells express RORC, also known as RAR-related orphan receptor gamma (RORγ or RORγt), which is a nuclear receptor crucial for the development and function of the Th17 lineage [84]. Elevated levels of RORC expression have been correlated with improved survival in patients with various cancers, including lung cancer, breast cancer, and esophageal adenocarcinoma, among others [102]. Xia et al. developed an RORγt agonist named 8-074, which specifically targets RORγt and promotes Th17 cell differentiation in both mice and humans [103]. Their research demonstrated that treatment with the 8-074 agonist resulted in significant antitumor effects across various tumor models. They observed an upregulation of IFN-γ+CD8+ T cell infiltration in tumors treated with the 8-074 RORγt agonist, driven by C-X-C Motif Chemokine Ligand 10 (CXCL10) produced by monocyte-derived dendritic cells (MoDCs). Combining the 8-074 RORγt agonist with anti-PD-1 treatment yielded superior efficacy compared to either treatment alone [103]. Similarly, the study by Tian et al. utilized another RORγt agonist, JG-1, which enhanced the differentiation of Th17 cells while inhibiting the differentiation of Treg cells [104]. JG-1 exhibited potent tumor growth inhibition across multiple syngeneic models and demonstrated synergistic effects when combined with anti-CTLA-4 antibody therapy. In tumors, JG-1 not only promoted Th17 cell differentiation and increased the expression of C-C Motif Chemokine Receptor 6 (CCR6) and CCL20 but also suppressed TGF-β expression and inhibited the differentiation and infiltration of Tregs. These findings underscore JG-1’s potent in vitro activity and significant in vivo antitumor effects, highlighting its potential as a therapeutic agent when combined with anti-CTLA-4 treatment [104].

In addition to IL-17, Th17 cells can produce other cytokines like IL-21 and IL-22, which may play significant roles in immune checkpoint inhibitor (ICI) therapy. In MIBC, the presence of IL-22+ Th cells within the tumor has been associated with poor prognosis and resistance to adjuvant chemotherapy (ACT). Notably, elevated levels of exhaustion markers—such as PD-1, CTLA-4, T-cell immunoglobulin and mucin domain (TIM3), and lymphocyte-activation gene 3 (LAG-3)—have been observed in CD8+ T cells within these tumors [104]. The authors demonstrated that intratumoral IL-22-producing T cells impaired the function of CD8+ T cells, while CD4+ T cells and NK cells remained unaffected [105]. To understand the clinical significance of IL-22-producing Th cell infiltration in the context of immunotherapy, the researchers conducted an experiment in which they added Nivolumab to freshly resected tumor tissue. They then measured the cytotoxic activity of CD8+ T cells and the apoptosis of tumor cells to assess how IL-22+ Th cells might influence the effectiveness of anti-PD-1 therapy [105]. The results showed an increase in the production of IFN-γ and granzyme B in CD8+ T cells, suggesting that patients with high infiltration of IL-22+ T cells might experience a better response to PD-1 therapy. This improvement was attributed to the fact that blocking IL-22 enhanced CD8+ T cell-dependent antitumor immunity. Consequently, targeting IL-22 could potentially augment the effectiveness of treatments such as Nivolumab. However, further clinical studies are necessary to confirm whether IL-22 expression can serve as a predictive biomarker for response to Nivolumab in patients with MIBC.

### Regulatory T cells

Regulatory T cells (Tregs) are suppressive T cells that preserve immune balance by inhibiting immune responses and ensuring tolerance to self tissues. When tolerance is not maintained, it can lead to development of autoimmune diseases and chronic inflammation [106]. Tregs include thymus-derived Tregs (tTregs), which mature in the thymus; peripheral Tregs (pTregs), which develop from naive T cells in peripheral tissues in response to tolerogenic signals such as interactions with immature dendritic cells; and inducible Tregs (iTregs), which are generated in vitro [107]. Regulatory T cells can inhibit immune responses through various contact-dependent and contact-independent mechanisms. In contact-dependent suppression, Tregs use surface molecules such as CTLA-4, PD-1, membrane-bound TGF-β, LAG-3, T cell immunoreceptor with immunoglobulin and ITIM domain (TIGIT), and galectin-9 (gal-9) to transmit negative signals to target cells. In contact-independent suppression, Tregs secrete suppressive cytokines like IL-10, TGF-β, and IL-35. Additionally, they can directly kill target cells, including T cells and antigen-presenting cells, by secreting perforin or granzyme B [108, 109].

Regulatory T cells are crucial for preventing autoimmunity and transplant rejection but can be detrimental to tumor immunity [13]. In 2001, Woo et al. demonstrated that Tregs were increased in patients with malignancies and suppressed T-cell activation in vitro [110]. Subsequent studies across various tumors confirmed that Tregs are present in the tumor microenvironment and promote tumor progression by inhibiting effective antitumor immunity [111–119]. Treg cells are attracted to tumors by chemokines produced either by the tumor itself or by immune cells infiltrating the tumor, such as tumor-associated macrophages (TAMs) or MDSCs [112–119]. They express chemokine receptors that enable their migration to cancerous lesions from various sites, including the thymus, lymph nodes, bone marrow, or peripheral blood [108, 110–118]. Upon arrival at the tumor site, Tregs exert immunosuppressive effects on cells with antitumor potential [120]. They secrete immunosuppressive factors such as TGF-β and IL-10, which enhance immunosuppression by reducing CD8+ T cell cytotoxicity, suppressing CD4+ T cell differentiation, promoting Treg conversion, and inhibiting NK cell proliferation [121]. Tregs also eliminate effector T cells and antigen-presenting cells via granzymes and perforins [122]. They interfere with normal immune cell metabolism by depriving CD4+ T cells of IL-2 [123] and hydrolyse ATP to produce adenosine, which inhibits effector cell proliferation [124]. Additionally, Tregs interact with DCs to suppress the immune response. By binding to DCs through CTLA-4, they inhibit T cell activation and induce DCs to express indoleamine 2,3-dioxygenase (IDO), which further suppresses T cell proliferation [125] and promotes the expansion of Tregs [126]. Moreover, Tregs foster an immunosuppressive microenvironment by interacting with MDSCs. They induce MDSCs to secrete IL-10 and TGF-β in response to IFN-γ. In turn, Tregs themselves secrete TGF-β and IL-35 to further enhance the suppressive functions of MDSCs [127, 128]. An in vitro study demonstrated that high numbers of Tregs impaired the effector function of tumor antigen-specific CD8+ T cells [129]. These compromised CD8+ T cells exhibited a naive phenotype, responded poorly to tumor antigen stimulation, had low proliferative capacity, and produced minimal IL-2 and other cytokines [130]. Research on Tregs isolated from tumor tissue has shown that these cells can induce the expression of other immune checkpoint inhibitors. When these receptors are upregulated, they block the activation of anti-tumor effector T cells, promoting Treg proliferation and contributing to tumor growth [131–133]. In many cancers, including ovarian, breast, gastric, and lung cancer, Treg infiltration of tumor tissue is associated with poorer patient survival [110, 134–136]. Similarly, research on peripheral Tregs has revealed a significant correlation with clinical outcomes. A higher frequency of peripheral blood Tregs has been proposed as a predictor of clinical responses to radiotherapy [137]. In a study involving 70 non-small-cell lung carcinoma (NSCLC) patients undergoing radiation therapy, elevated Treg levels independently predicted poorer progression-free survival [137]. However, evidence also suggests that the accumulation of Tregs is associated with improved prognosis in advanced-stage ovarian cancer and colorectal cancer patients undergoing chemotherapy or chemoimmunotherapy [138–141]. Shang et al. showed that FOXP3+ Tregs were associated with prolonged overall survival in head and neck and esophageal cancers, with no significant correlation observed in pancreatic cancer [135].

**Fig. 3:**
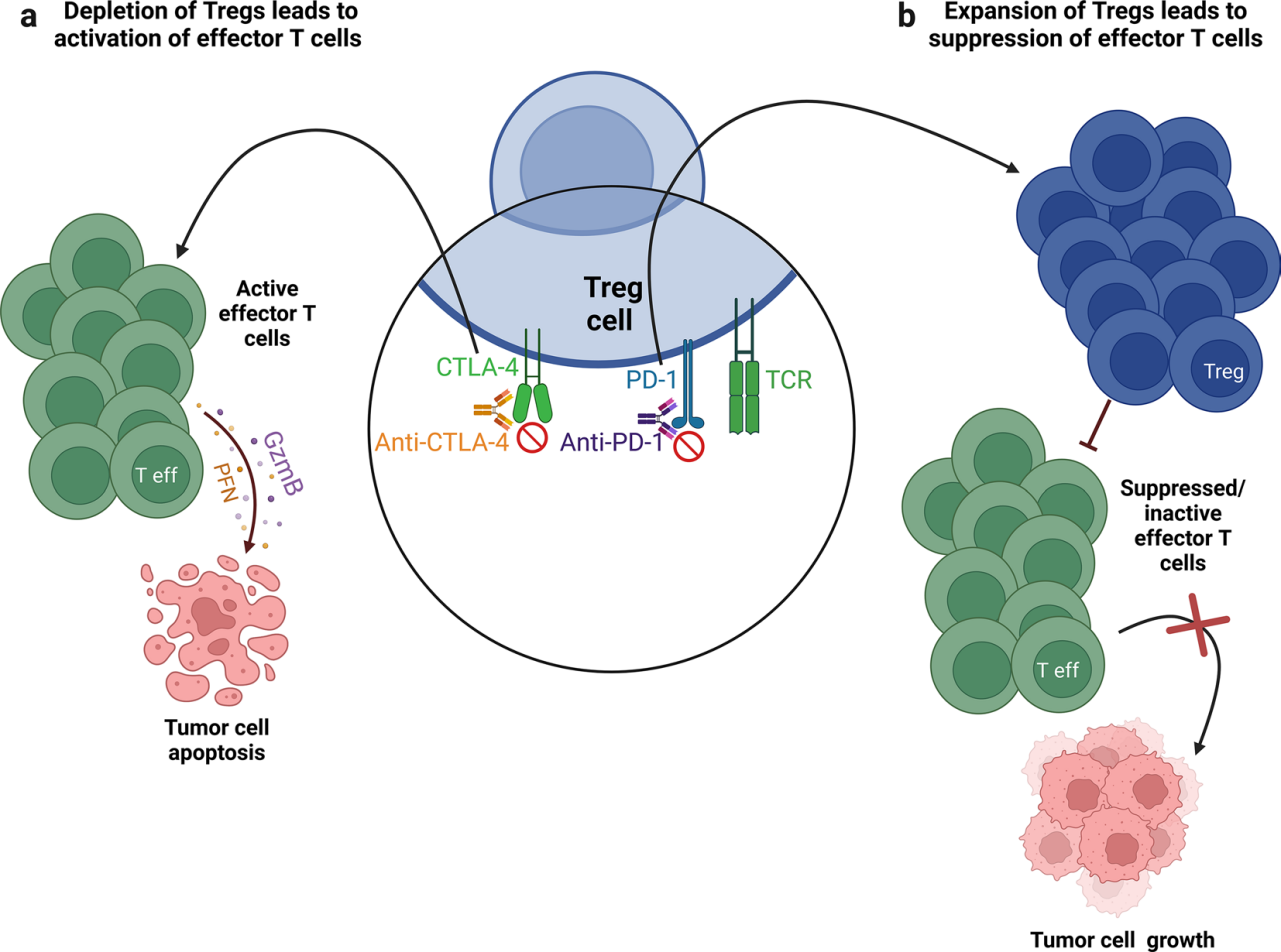
Effect of anti-CTLA-4 and anti-PD-1 antibodies on regulatory T cells. Regulatory T cells express CTLA-4 and PD-1 inhibitory receptors on their surface. Treatment with antibodies against CTLA-4 leads to the depletion of Tregs in the tumor microenvironment, resulting in the activation of effector T cells and subsequent apoptosis of tumor cells (**a**). In contrast, treatment with antibodies against PD-1 causes the proliferation of Tregs, which suppress effector T cells, leading to ineffective anti-tumor immunity and tumor growth (**b**). *Abbreviations TCR* t cell receptor, *CTLA-4* cytotoxic T-lymphocyte-associated antigen 4, *PD-1* programmed death 1, *Treg* regulatory T cell, *Teff* effector T cell, *GZMB* granzyme B, *PFN* perforin. The figure was prepared using Biorender

Tregs found abundantly within the tumor microenvironment not only suppress anti-cancer immune responses but also diminish the effectiveness of immune checkpoint inhibitor therapies [142]. These Tregs express higher levels of PD-1 inhibitory receptors compared to effector T cells and exhibit greater suppressive activity than their peripheral counterparts [106, 143]. Inhibiting Tregs is crucial for reducing their suppressive activity and enhancing anti-tumor immunity [144]. However, it appears that anti-PD-1 and anti-CTLA-4 antibodies have opposing effects on these cells (Fig. 3).

In some patients treated with Nivolumab, an unexpected outcome occurred where PD-1 inhibition led to an increase in the number of PD-1-expressing Tregs. These Tregs were highly suppressive, thereby more effectively dampening anti-tumor immune responses [143, 144]. Kamada et al. found that PD-1 blockade or deficiency enhanced the proliferation and immunosuppressive activity of PD-1+ Treg cells in humans. Approximately 10% of patients with advanced gastric cancer treated with anti-PD-1 antibodies experienced rapid disease progression, known as hyperprogressive disease (HPD), wherein PD-1-PD-L1 pathway blockade activated and expanded tumor-infiltrating PD-1+CTLA-4+ Tregs [145].

With the advent of immune checkpoint inhibitors in cancer treatment, there is considerable interest in identifying biomarkers that can predict therapeutic responses. Currently, PD-L1 expression on tumor cells is commonly used as a biomarker, with higher levels suggesting a better response to ICIs [146]. However, recent studies indicate that other factors may provide equally or more informative predictors of response to ICI treatment. One such factor is the presence of Tregs [147]. Koh et al. investigated peripheral Tregs as a potential biomarker for ICI responses. The study analyzed peripheral blood samples from 83 NSCLC patients before and after ICI-based immunotherapy. Patients with a high frequency of Tregs one week after anti-PD-1 administration showed significantly improved response rates and longer progression-free and overall survival. However, the number of peripheral Tregs prior to therapy did not predict outcomes [148]. Similar findings were observed for serum levels of TGF-β, which were also correlated with better clinical outcomes. These results were validated in a second cohort of 45 patients, suggesting that increased peripheral Tregs or elevated TGF-β levels can predict favorable clinical outcomes [148].

CTLA-4, a second inhibitory receptor, is constitutively expressed by Tregs and serves as a contact-dependent suppressive molecule crucial for maintaining immune tolerance [106, 108]. Studies have shown that Treg cells lacking CTLA-4 expression are unable to maintain their suppressive function, particularly failing to inhibit the expression of CD80 and CD86 on dendritic cells [149]. Importantly, the absence of CTLA-4 in Tregs also results in abnormal expression and proliferation of conventional T cells, which may infiltrate and potentially damage non-lymphoid tissues and cells [150]. Thus, CTLA-4 in Tregs is essential for preventing the accumulation of T cells that could harm critical organs.

Treg cells, characterized by high levels of CTLA-4 expression, are the primary FOXP3+CD4+ Treg cells infiltrating most types of tumors [106, 151–153]. The majority of studies indicate that anti-CTLA-4 antibodies primarily reduce Treg cell numbers in cancer [154]. The main mechanisms for Treg depletion are antibody-dependent cellular cytotoxicity (ADCC) and antibody-dependent cellular phagocytosis (ADCP) [151]. For instance, Ji et al. demonstrated that treatment with anti-CTLA-4 antibodies significantly reduced the population of CD25+FOXP3+ Treg cells in mice [155]. Qu et al. found that anti-CTLA-4 monoclonal antibodies enhanced the antitumor response stimulated by IL-36 by reducing Treg cells within the tumor microenvironment [156]. Kvarnhammar et al. showed that a bispecific antibody targeting both CTLA-4 and OX40 promoted T cell activation and depleted Treg cells both in vitro and in vivo within the tumor [157]. Moreover, Simson et al. observed a reduction in FOXP3+ Tregs within metastatic melanoma lesions following treatment with anti-CTLA-4 antibodies [158]. Similarly, research by Romano et al. indicated that Ipilimumab can activate CD16-expressing nonclassical monocytes ex vivo, leading to ADCC-mediated destruction of Tregs in metastatic melanoma. The study found that patients who responded to Ipilimumab had a reduction in Treg infiltration post-treatment compared to non-responders [159]. Du et al. also reported efficient depletion of Treg cells and Fc receptor-dependent tumor regression in mice treated with anti-CTLA-4 antibodies [160]. An interesting observation was made by Quezada et al., who investigated the effects of anti-CTLA-4 and a GM-CSF-transduced tumor cell vaccine (Gvax) on the balance between effector T cells (Teffs) and Tregs in a mouse model of melanoma [161]. Researchers observed that the introduction of the tumor increased the number of Tregs in the lymph nodes. Untreated tumors had infiltrates of both non-regulatory and CD4+FOXP3+ regulatory T cells, but only a few CD8+ T cells. Anti-CTLA-4 therapy did not deplete Tregs or permanently impair their function; instead, it acted intrinsically on both Tregs and Teffs, allowing them to expand. Gvax primed the tumor-reactive effector T cells, leading to their activation, infiltration into the tumor, and a delay in tumor growth. Combining Gvax with CTLA-4 blockade resulted in increased infiltration of effector T cells into the tumor and a significant shift in the balance between Tregs and effector T cells (Teffs) within the tumor. This shift in the intratumoral ratio of Tregs to Teffs directly correlated with tumor rejection [161].

However, fewer studies have reported no effect of anti-CTLA-4 blockade on Tregs, or even an increase in Treg cell counts [154, 162–166].

When discussing the negative impact of immune checkpoint inhibitors (ICIs) on Tregs, it is worth mentioning the recent findings of Green et al., who discovered a subset of CD29+ regulatory T cells that respond to ICIs and are highly suppressive [167]. Considering that liver metastases from various primary tumors often show poor responses to ICIs [168], the authors explored the impact of immune checkpoint blockade therapy in mouse models of liver metastasis. They observed that the therapy failed to inhibit tumor growth when tumors were located in the liver, although it was effective against subcutaneous tumors. Further analysis of liver immune cells revealed an unexpected increase in the proliferation and accumulation of CD29-expressing regulatory T cells, along with the upregulation of activation markers on their surface, after treatment with various ICIs [167]. These CD29-positive regulatory T cells were also identified within the tumor microenvironment of human liver cancer, where CD29 expression correlated with Treg proliferation in both humans and mice. Consequently, the authors propose exploring the combination of ICIs with CD29-targeting agents to counteract Treg-mediated resistance in liver tumors [167].

Based on current knowledge, while anti-CTLA-4 antibodies are designed to enhance antitumor immune responses by inhibiting CTLA-4-mediated suppression of effector T cells, their impact on Tregs can be variable and context-dependent. Further research is needed to fully understand these effects and to optimize therapeutic strategies involving these immune checkpoint inhibitors. A promising strategy involves combining PD-1 and CTLA-4 blockers to enhance antitumor immunity. This approach could simultaneously relieve effector T cells from PD-1/PD-L1-mediated exhaustion and deplete intratumoral Tregs. Research supports this, showing that the combination of Ipilimumab and Nivolumab significantly improves treatment efficacy in patients with metastatic melanoma and is likely more effective in other cancers compared to using these antibodies as monotherapies [169, 170]. Similarly, combining antibodies that target CD25 with anti-PD-1 treatment is expected to enhance the activity of effector CD4+ and CD8+ T cells [171]. Additionally, using anti-PD-1 antibodies conjugated with the enzyme adenosine deaminase, which converts adenosine to inosine, or bispecific fusion proteins targeting PD-(L)1 and TGF-β could help reduce Tregs and revitalize tumor-specific effector T cells [171, 172].

## Summary and future directions

Th subsets play critical roles in tumor immunity: Th1 cells generally promote anti-tumor responses through IFN-γ production, while Th2 cells and Tregs often support tumor growth by suppressing effective immune responses. Th9 and Th17 cells exhibit dual roles with context-dependent effects on tumor progression. A detailed investigation into the molecular and cellular mechanisms by which each Th subset influences tumor immunity, including their signaling pathways, cytokine profiles, and interactions with other immune cells, is crucial for developing more precise and effective immunotherapies. Immune checkpoint inhibitors enhance anti-tumor responses by modulating Th subsets, typically boosting Th1 activity and often downregulating Treg-mediated suppression. Future research should focus on developing biomarkers to identify and quantify the activity of different Th subpopulations in patients, predicting responses to ICIs and tailoring personalized treatment plans. Additionally, investigating agents that specifically modulate the activity of Th subsets and designing combination therapies—such as combining PD-1 inhibitors with agents that block Treg activity (e.g., anti-CD25 or anti-CTLA-4 antibodies)—are crucial for enhancing the overall anti-tumor immune response.

Moreover, conducting detailed mechanistic studies to understand how Th subsets interact with ICIs, including exploring the pathways and cytokines involved in these interactions and how they influence tumor immunity, is essential. Research should also focus on the tumor microenvironment and its effects on Th subpopulations’ differentiation and function, as well as the plasticity of Th cells, or how Th cells can switch between subsets. Understanding these dynamics can help develop strategies to manipulate the tumor microenvironment to favor anti-tumor Th responses. Exploring how Th cell modulation can be integrated with other cancer therapies, such as chemotherapy, radiotherapy, and targeted therapies, is another important area. Finally, developing more sophisticated preclinical models that better mimic human Th subset dynamics and tumor interactions is crucial for testing hypotheses and therapeutic approaches before clinical application. This comprehensive understanding of Th subsets can guide the design of various treatments that enhance the anti-tumor activity of beneficial Th subsets while mitigating the suppressive effects of others, ultimately improving the efficacy and safety of immune checkpoint inhibitors and leading to more personalized cancer treatment strategies.

## Data Availability

No datasets were generated or analysed during the current study.
